# Effects of a Personalized Smartphone App on Bowel Preparation Quality: Randomized Controlled Trial

**DOI:** 10.2196/26703

**Published:** 2021-08-19

**Authors:** Quirine E W van der Zander, Ankie Reumkens, Bas van de Valk, Bjorn Winkens, Ad A M Masclee, Rogier J J de Ridder

**Affiliations:** 1 Division of Gastroenterology and Hepatology Department of Internal Medicine Maastricht University Medical Center Maastricht Netherlands; 2 GROW, School for Oncology and Developmental Biology Maastricht University Maastricht Netherlands; 3 NUTRIM, School of Nutrition and Translational Research in Metabolism Maastricht University Maastricht Netherlands; 4 Department of Methodology and Statistics Maastricht University Maastricht Netherlands

**Keywords:** colonoscopy, laxatives, bowel preparation, smartphone application, smartphone, patient satisfaction, randomized controlled trial, mobile phone, mHealth

## Abstract

**Background:**

Adequate bowel preparation is essential for the visualization of the colonic mucosa during colonoscopy. However, the rate of inadequate bowel preparation is still high, ranging from 18% to 35%; this may lead to a higher risk of missing clinically relevant lesions, procedural difficulties, prolonged procedural time, an increased number of interval colorectal carcinomas, and additional health care costs.

**Objective:**

The aims of this study are to compare bowel preparation instructions provided via a personalized smartphone app (Prepit, Ferring B V) with regular written instructions for bowel preparation to improve bowel preparation quality and to evaluate patient satisfaction with the bowel preparation procedure.

**Methods:**

Eligible patients scheduled for an outpatient colonoscopy were randomized to a smartphone app group or a control group. Both the groups received identical face-to-face education from a research physician, including instructions about the colonoscopy procedure, diet restrictions, and laxative intake. In addition, the control group received written information, whereas the smartphone app group was instructed to use the smartphone app instead of the written information for the actual steps of the bowel preparation schedule. All patients used bisacodyl and sodium picosulfate with magnesium citrate as laxatives. The quality of bowel preparation was scored using the Boston Bowel Preparation Scale (BBPS) by blinded endoscopists. Patient satisfaction was measured using the Patient Satisfaction Questionnaire-18.

**Results:**

A total of 87 patients were included in the smartphone app group and 86 in the control group. The mean total BBPS score was significantly higher in the smartphone app group (mean 8.3, SD 0.9) than in the control group (mean 7.9, SD 1.2; *P*=.03). The right colon showed a significantly higher bowel preparation score in the smartphone app group (mean 2.7, SD 0.5 vs mean 2.5, SD 0.6; *P*=.04). No significant differences were observed in segment scores for the mean transverse colon (mean 2.8, SD 0.4 vs mean 2.8, SD 0.4; *P*=.34) and left colon (mean 2.8, SD 0.4 vs mean 2.6, SD 0.5; *P*=.07). General patient satisfaction was high for the smartphone app group (mean 4.4, SD 0.7) but showed no significant difference when compared with the control group (mean 4.3, SD 0.8; *P*=.32).

**Conclusions:**

Our personalized smartphone app significantly improved bowel preparation quality compared with regular written instructions for bowel preparation. In particular, in the right colon, the BBPS score improved, which is of clinical relevance because the right colon is considered more difficult to clean and the polyp detection rate in the right colon improves with improvement of bowel cleansing of the right colon. No further improvement in patient satisfaction was observed compared with patients receiving regular written instructions.

**Trial Registration:**

ClinicalTrials.gov NCT03677050; https://clinicaltrials.gov/ct2/show/NCT03677050

## Introduction

### Background

Colonoscopy is considered the gold standard for diagnosing colorectal pathologies. The efficacy and safety of colonoscopy are related to the quality of the preinvestigational bowel preparation. Adequate bowel preparation is essential for the optimal visualization of the colonic mucosa during colonoscopy. Inadequate bowel preparation is associated with the risk of missing clinically relevant lesions, procedural difficulties, prolonged procedural time, an increased number of interval colorectal carcinomas, and additional health care costs [1-6]. Currently reported rates of inadequate bowel preparation range from 18% to 35% [1,7], leaving room for improvement.

Previous studies have evaluated various factors that can negatively affect bowel preparation, such as dietary restrictions (low-fiber vs clear liquid diet), laxative administration (single vs split dose), inadequate information precolonoscopy, and long waiting times [8-12]. In addition, bowel preparation quality depends on patients’ tolerability to the laxative and patients’ satisfaction. Patient satisfaction is inherently correlated with patients’ compliance with the physician-recommended bowel preparation schedules.

Strategies to improve bowel preparation aim to inform patients more extensively about the preparation procedure and remind patients when action is needed (ie, start of diet modifications and intake of the laxative). Several of these strategies, including visual aids, educational videos, and SMS reminders, have provided better bowel preparation quality when compared with regular instructions [13]. Current colonoscopy preparation guidelines recommend providing patients with both verbal and written instructions and acknowledge the added value of providing educational booklets [14,15].

### Objectives

A new method for informing and instructing patients is via a personalized smartphone app. In 2017, 93% of Dutch adults possessed a smartphone. The highest percentage of smartphone use was found in the younger age groups, but 90% of people aged ≥55 years had access to a smartphone [16]. Therefore, this technology has the potential to improve bowel preparation quality during colonoscopy. This study aims to investigate the quality of bowel preparation and patient satisfaction in patients using a newly developed, personalized smartphone app in addition to verbal instructions compared with regular verbal and written instructions.

## Methods

### Study Design

This prospective, endoscopist-blinded, randomized controlled trial was conducted at the Maastricht University Medical Center+, Maastricht, the Netherlands, from August 2018 to November 2019. The study was conducted in accordance with the Declaration of Helsinki [17] and the General Data Protection Regulation [18]. The Medical Ethical Review Committee of the Maastricht University Medical Center (MEC 16-4-141) approved the study. This study is registered at ClinicalTrials.gov (NCT03677050).

### Subjects

Patients who were aged ≥18 years, who possessed a smartphone, who were referred to the outpatient clinic for a colonoscopy screening visit by their general practitioner or by the Dutch colorectal cancer screening program, and who were prescribed sodium picosulfate with magnesium citrate (SPMC) were eligible to participate. Hospitalized patients, patients undergoing an emergency colonoscopy, and patients without a smartphone were not considered eligible for participation. All patients fulfilling these inclusion and exclusion criteria were considered for inclusion in this study, and all included patients provided written informed consent. No incentives were offered to participating patients.

### Randomization and Group Description

**Figure 1:**
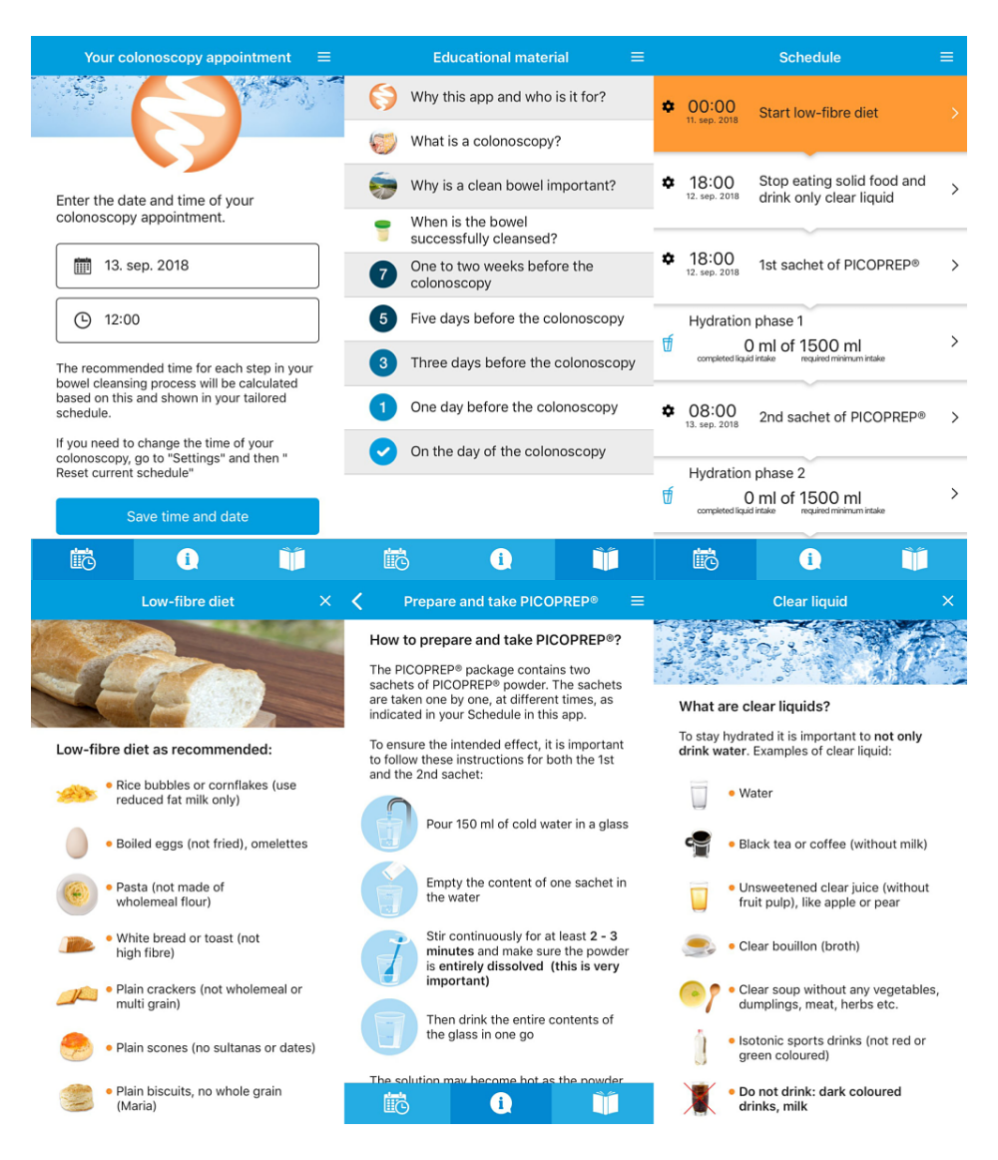
Smartphone app screenshots. (A) date and time entry, (B) educational tools, (C) date and time specific bowel preparation schedule, (D) examples of low-fiber diet, (E) picoprep preparation instructions, and (F) examples of clear liquids. Copyright Prepit, Ferring B V.

Patient education occurred during a screening visit at the outpatient clinic 1-4 weeks before colonoscopy. During this visit, patients were randomly assigned to the smartphone app group or the control group using a computer-generated randomization list in a 1:1 sequence based on the order of inclusion. Patients from both the groups received a hyperlink to a web-based educational video explaining the colonoscopy procedure. Patients in the control group received verbal and written information concerning diet restrictions, bowel preparation schedules, and laxatives. Patients in the smartphone app group had to install the app on their Android or iOS smartphones, which was accessible by a quick response code (Prepit, Ferring B V; for the Consolidated Standards of Reporting Trials, see Multimedia Appendix 1 [1,7,16,19-25]). Instead of written instructions, patients in the smartphone app group received information and instructions via the smartphone app. The information and instructions provided via the smartphone app were similar to the written instructions of the control group. However, the information was presented in a more visual way, that is, providing pictograms of low-fiber food products and images of the desired stool consistency after ingestion of the laxatives. Furthermore, the smartphone app provided the patients with personalized notifications about the steps of bowel preparation tailored to the exact colonoscopy date and time (Figure 1). It did not take extra time to provide the explanation via the smartphone app compared with the explanation given via the written instructions. Patient satisfaction with the bowel preparation procedure was evaluated using a self-assessed paper questionnaire, the Patient Satisfaction Questionnaire-18 (PSQ-18). This questionnaire was handed out to the patients during the screening visit and filled in by the patients on the day of the colonoscopy. Patients completed the questionnaire before the colonoscopy was performed, as the actual experience of undergoing the colonoscopy was not asked for and could possibly (both negatively and positively) influence patient satisfaction regarding the bowel preparation procedure (for the questionnaire, please refer to Multimedia Appendix 2 [26]).

### Bowel Preparation Schedule and Instructions

Instructions were delivered face-to-face by 2 research physicians (QEWVDZ and BVDV). Patients were instructed to follow a low-fiber diet 2 days before the colonoscopy. All patients were prescribed SPMC in a split-dose regimen of 2 doses, consisting of 10.0 mg sodium picosulfate, 3.5 g magnesium oxide, and 12.0 g citric acid (Picoprep, Ferring B V). Patients scheduled for a colonoscopy in the morning or early afternoon were instructed to take the first SPMC dose the evening before and the second dose the morning of the colonoscopy. For colonoscopies scheduled in the afternoon, patients had to take both SPMC doses the morning of the examination, with a 2- to 5-hour interval between both the doses. All patients were also administered 10.0 mg of bisacodyl as an additive to the first SPMC dose.

### Outcomes

The primary outcome was bowel preparation quality assessed using the Boston Bowel Preparation Scale (BBPS). The BBPS is a validated and reliable scale that rates bowel cleanliness for each colonic segment (right, transverse, and left) after washing, suctioning, and cleaning maneuvers have been performed by the endoscopist [27]. Each segment is scored on a scale from 0 to 3 (3 being the cleanest) [28,29]. Segment scores were summed to calculate the total BBPS, which ranged from 0 to 9. Bowel preparation was considered adequate when the total score was ≥6 and all segment scores were ≥2. This cut-off value has been shown to be adequate for detecting polyps >5 mm [28-30]. The endoscopists were blinded to the study groups. Secondary end points were adenoma detection rate (ADR), polyp detection rate (PDR), cecal intubation time, and withdrawal time. ADR and PDR were calculated by dividing the number of patients with at least one adenoma and one polyp, respectively, by the total number of colonoscopy patients (based on the histological diagnosis according to the revised Vienna classification) [19,20]. Withdrawal time included the time from starting withdrawal from the cecum to the final inspection of the rectum, including the time spent on washing, suctioning, and polypectomies.

Items from the PSQ-18 were transformed to bowel preparation education purposes to investigate patient satisfaction [26]. Scores for the following subscales were calculated by averaging the scores of the relevant questions: general satisfaction (items 3 and 6), technical quality (items 8 and 9), communication (items 1 and 2), time spent on education (item 7), and convenience (items 4 and 5). Responses to all items were given on a five-point Likert scale, ranging from strongly agree to strongly disagree. Patients in the smartphone app group were also asked to rate the user friendliness and design of the smartphone app on a 10-point scale.

### Statistical Analysis and Sample Size

Sample size calculation was performed using PS Power and Sample Size Program version 3.1.2 (W D Dupont and W D Plummer, Jr). To detect a difference of 0.75 in the total BBPS scores between both groups with a significance level (*P* value) of .05 and a power of 80%, 82 completers per group were needed [21,22]. To account for patients dropping out, 90 patients per group were enrolled.

Intention-to-treat analyses were performed. Descriptive statistics are presented as mean (SD) or as the number of patients (%). Differences between study groups were analyzed using two-tailed independent-samples *t* test for numerical variables and chi-square test or Fisher exact test for categorical variables. Posthoc analyses were performed for subgroup analyses. Two-sided *P* values ≤.05 were considered statistically significant. Statistical analyses were performed using SPSS Statistics for Windows, version 25 (IBM).

## Results

### Study Population

**Figure 2:**
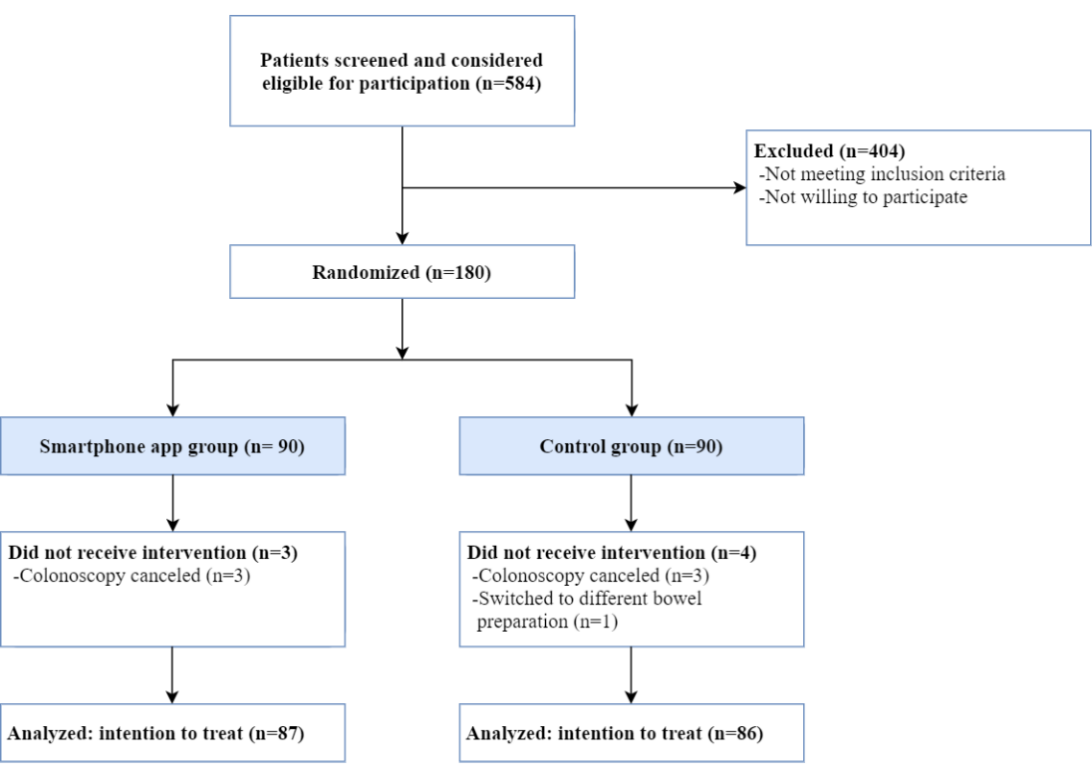
Study flowchart of patient enrollment and inclusion.

Patients who underwent a colonoscopy at the Maastricht University Medical Center+ between August 2018 and November 2019 were screened for eligibility. In total, 90 patients were included in the smartphone app group and 90 in the control group (Figure 2). A total of 7 patients were excluded from the study. Patient characteristics are provided in Table 1. No significant differences were observed between the smartphone app group and the control group in terms of baseline characteristics. Patients in both the groups had the same level of experience in using medical smartphone apps.

**Table 1:** Baseline characteristics of patients in the smartphone app group and patients in the control group.

Baseline characteristics			Smartphone app group (N=87)		Control group (N=86)		*P* value	
**Age (years)**								
	Value, mean (SD)	56.9 (10.8)		57.1 (12.4)		.92		
	Age<65, n (%)	67 (77)		62 (72)		.46		
	Age≥65, n (%)	20 (23)		24 (28)		.46		
Gender, female, n (%)			37 (43)		34 (40)		.69	
BMI in kg/m^2^, mean (SD)			26.1 (4.6)		25.7 (3.6)		.56	
**Indication for colonoscopy, n (%)**								.25
	National screening program	29 (33)		21 (24)				
	Surveillance	17 (20)		25 (29)				
	Symptoms	41 (47)		40 (47)				
Waiting time in days, mean (SD)^a^			26.8 (17.6)		31.6 (24.6)		.14	
Previous colonoscopy, n (%)			34 (39)		37 (43)		.60	
**Gastrointestinal history, n (%)^b^**			37 (43)		43 (50)		.32	
	Diverticulosis	10 (11)		16 (19)		.19		
	Constipation	14 (16)		18 (21)		.41		
	Abdominal or pelvic surgery^c^	22 (25)		16 (19)		.28		
Comorbidities, n (%)^d^			45 (52)		37 (43)		.25	
**Level of education, n (%)**								.37
	High school	15 (20)		9 (13)				
	Secondary vocational education	28 (37)		24 (34)				
	Higher education (including Bachelor and Master programs at universities of applied sciences)	33 (43)		38 (54)				
**Experienced in using smartphone apps, n (%)**			76 (99)		59 (86)		*.*003	
	More than 10 apps	52 (69)		43 (73)		.65		
Previous medical smartphone app use, n (%)			7 (9)		8 (12)		.62	

^a^Waiting time was defined as the time between screening visit and colonoscopy.

^b^Inflammatory bowel disease and stenosis did not occur in any patients’ medical history.

^c^Abdominal or pelvic surgery included colectomy, abdominal uterus extirpation, prostatectomy, appendectomy, nephrectomy, cholecystectomy, and cesarean delivery.

^d^Comorbidities included hypertension, cardiovascular disease, chronic pulmonary disease, renal disease, liver disease, psychiatric disease, and diabetes mellitus.

### Bowel Preparation Quality

Colonoscopies were performed by 25 different endoscopists (gastroenterologists and fellows) who rated the BBPS. All endoscopists were experienced in scoring the BBPS. The mean total BBPS score in the smartphone app group was significantly higher than that in the control group (mean 8.3, SD 0.9 vs mean 7.9, SD 1.2; *P*=.03). Mean right colon segment scores were also significantly higher in the smartphone app group (mean 2.7, SD 0.5 vs mean 2.5, SD 0.6; *P*=.04). No significant differences were observed in the mean transverse colon and left colon segment scores (Table 2). One patient in the smartphone app group and 4 patients in the control group had inadequate bowel preparation scores (*P*=.18). Multivariable logistic regression analyses, to reveal independent predictors for inadequate bowel preparation, could not be performed because of this low number.

**Table 2:** Bowel preparation scores for the smartphone app group and the control group.^a^

Bowel preparation quality		Smartphone app group (n=81)	Control group (n=81)	*P* value
**BBPS,^b^ mean (SD)**				
	Total	8.3 (0.9)	7.9 (1.2)	*.03* ^c^
	BBPS right colon	2.7 (0.5)	2.5 (0.6)	*.04*
	BBPS transverse colon	2.8 (0.4)	2.8 (0.4)	.34
	BBPS left colon	2.8 (0.4)	2.6 (0.5)	.07
**Adequate bowel preparation, n (%)^d^**		80 (99)	77 (95)	.18^e^
	Total BBPS score ≥6	81 (100)	79 (98)	.25^e^
	All segment scores ≥2	80 (99)	77 (95)	.18^e^

^a^Analyses for the Boston Bowel Preparation Scale included only complete colonoscopies (successful cecal intubation). Missing data were equally distributed between the smartphone app group (n=5) and the control group (n=5). Analyses including incomplete colonoscopies showed similar results.

^b^BBPS: Boston Bowel Preparation Scale.

^c^Italicization represents statistically significant result (*P*<.05).

^d^Adequate bowel preparation was defined as a total Boston Bowel Preparation Scale score of ≥6 and segment scores of ≥2.

^e^Fisher exact test.

Subgroup analyses were performed for morning and afternoon colonoscopies, age below and above 65 years, and colonoscopy waiting time exceeding 1 month or not (because of an increased risk of forgetting preparation instructions over time; Table 3). These analyses showed that patients aged <65 years in the smartphone app group had a significantly higher mean total (mean 8.4, SD 0.9 vs mean 7.9, SD 1.1; *P*=.01) and right BBPS score (mean 2.8, SD 0.4 vs mean 2.5, SD 0.6; *P*=.01) than those in the control group. Patients in the smartphone app group having an afternoon colonoscopy also had a significantly higher mean total and right BBPS score than those in the control group. Furthermore, patients with a colonoscopy waiting time >1 month in the smartphone app group had a significantly higher mean total BBPS score and a significantly cleaner left colon than those in the control group. No significant differences were observed for morning colonoscopies, age ≥65 years, and colonoscopies performed within 1 month.

**Table 3:** Subgroup analysis for the smartphone app group and the control group.^a^

Subgroup analyses		Smartphone app group (n=81)	Control group (n=81)	*P* value
**Afternoon colonoscopy**				
	Patient, n (%)	37 (46)	35 (43)	.75^b^
	Total BBPS,^c^ mean (SD)	8.3 (1.0)	7.7 (1.3)	.03
	BBBS right colon, mean (SD)	2.7 (0.5)	2.4 (0.6)	.04
	BBPS transverse colon, mean (SD)	2.8 (0.4)	2.7 (0.5)	.50
	BBPS left colon, mean (SD)	2.8 (0.4)	2.5 (0.6)	.02
**Morning colonoscopy**				
	Patient, n (%)	44 (54)	46 (57)	.75^b^
	Total BBPS, mean (SD)	8.3 (0.9)	8.1 (1.0)	.37
	BBBS right colon, mean (SD)	2.7 (0.5)	2.6 (0.5)	.38
	BBPS transverse colon, mean (SD)	2.8 (0.4)	2.8 (0.4)	.49
	BBPS left colon, mean (SD)	2.8 (0.4)	2.7 (0.5)	.73
**Age <65 years**				
	Patient, n (%)	63 (78)	57 (70)	.28^b^
	Total BBPS, mean (SD)	8.4 (0.9)	7.9 (1.1)	.01
	BBBS right colon, mean (SD)	2.8 (0.4)	2.5 (0.6)	.01
	BBPS transverse colon, mean (SD)	2.8 (0.4)	2.7 (0.4)	.17
	BBPS left colon, mean (SD)	2.8 (0.4)	2.7 (0.5)	.14
**Age ≥65 years**				
	Patient, n (%)	18 (22)	24 (30)	.28^b^
	Total BBPS, mean (SD)	7.9 (1.0)	7.9 (1.3)	.94
	BBBS right colon, mean (SD)	2.4 (0.6)	2.5 (0.6)	.61
	BBPS transverse colon, mean (SD)	2.7 (0.5)	2.8 (0.4)	.61
	BBPS left colon, mean (SD)	2.7 (0.5)	2.6 (0.5)	.37
**Colonoscopy waiting time >1 month**				
	Patient, n (%)	27 (33)	36 (44)	.15^b^
	Total BBPS, mean (SD)	8.3 (0.8)	7.7 (1.1)	.02
	BBBS right colon, mean (SD)	2.6 (0.6)	2.4 (0.6)	.31
	BBPS transverse colon, mean (SD)	2.9 (0.4)	2.7 (0.5)	.21
	BBPS left colon, mean (SD)	2.9 (0.4)	2.5 (0.5)	.004
**Colonoscopy waiting time ≤1 month**				
	Patient, n (%)	54 (67)	45 (56)	.15^b^
	Total BBPS, mean (SD)	8.3 (1.0)	8.1 (1.2)	.38
	BBBS right colon, mean (SD)	2.7 (0.5)	2.6 (0.6)	.12
	BBPS transverse colon, mean (SD)	2.8 (0.4)	2.8 (0.4)	.83
	BBPS left colon, mean (SD)	2.7 (0.4)	2.7 (0.5)	.94

^a^Analyses for the Boston Bowel Preparation Scale included only complete colonoscopies (successful cecal intubation). Missing data were equally distributed between the smartphone app group (n=6) and the control group (n=5). Analyses including incomplete colonoscopies showed similar results.

^b^Chi-square test comparing presence in specific subgroups (afternoon vs morning, age <65 years vs age ≥65 years, and colonoscopy waiting time ≤1 month vs colonoscopy waiting time >1 month) between the smartphone app group and the control group.

^c^BBPS: Boston Bowel Preparation Scale.

### Colonoscopy Quality Parameters

The cecal intubation rate was 93% and 94% in the smartphone app group and the control group, respectively (*P*=.77; Table 4). Eleven colonoscopies were incomplete because of severe pain sensations (n=6), stenosis (n=3), and technical difficulties (n=2). No colonoscopies were aborted because of inadequate bowel preparation. The mean withdrawal time did not differ significantly between the smartphone app group and the control group (Table 4). Both ADR and PDR were higher in patients in the smartphone app group than in patients in the control group, but the difference was not statistically significant.

**Table 4:** Colonoscopy quality parameters for the smartphone app group and the control group.

Colonoscopy quality parameters	Smartphone app group (n=87)	Control group (n=86)	*P* value
Cecal intubation rate, n (%)	81 (93)	81 (94)	.77
Withdrawal time in minutes, mean (SD)^a^	15.8 (8.6)	14.0 (9.1)	.20
Adenoma detection rate, n (%)^b^	35 (43)	27 (33)	.20
Polyp detection rate, n (%)^b^	44 (54)	36 (44)	.20

^a^Analyses for withdrawal time included only complete colonoscopies (successful cecal intubation). Withdrawal time could not be calculated for n=3 in the smartphone app group and not for n=1 in the control group.

^b^Analyses for adenoma and polyp detection rate included only complete colonoscopies (successful cecal intubation). Missing data were equally distributed between the smartphone app group (n=6) and the control group (n=5). Analyses including incomplete colonoscopies showed similar results.

### Patient Satisfaction

The response rates of the PSQ-18 were 85% (74/87) in the smartphone app group and 83% (71/86) in the control group (*P*=.66). On a five-point Likert scale, the general satisfaction was 4.4 (SD 0.7) in the smartphone app group and 4.3 (SD 0.8) in the control group (*P*=.32). No significant differences in patient satisfaction were observed in terms of technical quality, communication, time spent on education, and convenience (Table 5). The majority of smartphone app users were willing to use the app again for eventual future colonoscopies (mean 4.5, SD 0.6) and rated the added value of the smartphone app 4.4 (SD 0.7). On a 10-point scale, user friendliness and design of the smartphone app were rated 8.7 (SD 1.1) and 8.7 (SD 1.2), respectively.

**Table 5:** Patient satisfaction according to the Patient Satisfaction Questionnaire-18 and patient satisfaction with smartphone app use.^a^

Patient satisfaction			Smartphone app group (n=74)		Control group (n=71)		*P* value
**PSQ-18^b^ (5-point scale), mean (SD)**							
	General satisfaction	4.4 (0.7)		4.3 (0.8)		.32	
	Technical quality	4.5 (0.7)		4.5 (0.6)		.70	
	Communication	4.6 (0.5)		4.7 (0.6)		.52	
	Time spent on education	4.6 (0.7)		4.7 (0.6)		.45	
	Convenience	4.4 (0.7)		4.5 (0.6)		.45	
**Patient satisfaction on smartphone app use (5-point scale),^c^ mean (SD)**							
	Added value of the smartphone app	4.4 (0.7)		N/A^d^		N/A	
	Willingness to use the app for future colonoscopies	4.5 (0.6)		N/A		N/A	
	Ease of downloading and using	4.6 (0.7)		N/A		N/A	
	Clear overview of times to use laxative	4.6 (0.7)		N/A		N/A	
**Patient satisfaction on smartphone app use (10-point scale),^c^ mean (SD)**							
	Ease of use in general	8.7 (1.1)		N/A		N/A	
	Design	8.7 (1.2)		N/A		N/A	

^a^Analyses for patient satisfaction included only complete questionnaires. Analyses including incomplete questionnaires showed similar results.

^b^PSQ-18: Patient Satisfaction Questionnaire-18.

^c^Analyses for patient satisfaction on smartphone app use was only applicable for smartphone app users and based on n=78 complete questionnaires.

^d^N/A: not applicable.

## Discussion

### Principal Findings

Adequate bowel preparation is an important quality indicator for colonoscopy. The key finding of this study is the significantly higher mean total BBPS score in patients using a personalized smartphone app for bowel preparation instructions compared with patients using regular verbal and written information. Patient satisfaction did not improve further for smartphone app users compared with patients receiving regular written instructions.

### Comparison With Previous Work

The finding of a significantly higher mean total BBPS score in the smartphone app group compared with the control group is in line with previous studies [2,31,32]. The mean total BBPS score in the control groups of these studies ranged from 5.8 to 7.2. Although the mean total BBPS score (mean 7.9, SD 1.2) in our control group was high, the smartphone app still had added value (mean total BBPS score 8.3, SD 0.9). In particular, the mean BBPS score of the right colon was significantly higher in the smartphone app group than in the control group. This finding is clinically relevant because the right colon is considered more difficult to clean [33] and the PDR in the right colon improves with improvement in BBPS score of the right colon [34].

The European Society of Gastrointestinal Endoscopy recommends the use of enhanced instructions for bowel preparation. Methods such as telephone calls, visual aids, educational videos, and SMS reminders help to improve bowel preparation quality compared with regular instructions [1,2,4,13,33,35-37]. Possible advantages of smartphone apps are that they are more easily understandable, accessible, and interactive. Another benefit is that automatic alerts, reminders, and notifications remind patients to start and adhere to the steps of the bowel preparation schedule more precisely [38,39] without consuming valuable time and resources, as is the case with telephone calls [13,35], making smartphone apps easier to implement in daily clinical practice. Furthermore, the smartphone app provided a personalized bowel preparation schedule for each patient. The different steps of the bowel preparation procedure were adapted to the exact date and time of colonoscopy. In contrast, written instructions were general for morning and afternoon colonoscopies and indicated no exact date.

Previous studies included relatively young patients with a mean age of 42-55 years [2,21,35,36]. In this study, no maximum age for participation was stated, so older age groups, who might be less familiar with smartphone apps, were also included. Jeon et al [37] used a smartphone mobile messenger to educate patients and found that this approach was useful with respect to the quality of bowel preparation for the younger age group (<40 years) but not for patients aged >40 years. In our study, subgroup analysis showed significantly higher total mean BBPS scores and right colon segment scores for patients aged <65 years using the smartphone app compared with the control group. In addition to the study by Jeon et al [37], the significantly higher mean BBPS scores indicate that the use of a smartphone app is a feasible method not only for patients aged <40 years but also for patients aged <65 years. For patients aged ≥65 years, no significant differences in mean BBPS scores were found, although their number was low. Further research focusing on older patients (≥65 years) is needed to investigate the usefulness of a smartphone app among these patients.

In this study, the BBPS was used to measure bowel cleansing. A systematic review by Parmar et al [27] revealed that the BBPS is the most thoroughly validated scale and should therefore be used in clinical practice. It should be noted that the BBPS is scored after appropriate washing and suctioning steps have been performed. Therefore, differences in initial bowel preparation could have been masked by variations in the extent of the endoscopists’ washing and suctioning actions. However, because blinded endoscopists performed colonoscopies in both groups, potential differences in the extent of washing and suctioning were eliminated.

The minimum standard rate for adequate bowel preparation of ≥90%, a set criterion by the European Society of Gastrointestinal Endoscopy guidelines [40], was reached in both the smartphone app group and the control group. In 5 patients (5/173, 2.9%), the colon was inadequately prepared. In the literature, the reported numbers are higher, up to 35% [7,10,13,35]. In this study, predictors for inadequate bowel preparation could not be identified because of the low number of patients. In two meta-analyses, three groups of predictors for inadequate bowel preparation were identified: patients’ characteristics (increasing age, male gender, and higher BMI), clinical conditions (constipation, diabetes mellitus, hypertension, cirrhosis, stroke, and dementia), and medication use (narcotics and tricyclic antidepressants) [41,42]. Other studies also reported low level of education, low socioeconomic status, low health literacy, and low patient motivation in health promotion as influencing factors [13,35].

ADR and cecal intubation rate are indicators of colonoscopy quality [30]. Guo et al [43] found a significantly higher ADR in the smartphone app group than in the control group (21.4% vs 12.8%, respectively; *P*=.03). Although higher ADR and PDR were observed in the smartphone app group in this study, the observed differences were not statistically significant. It should be noted that this study was not powered to detect significant differences in ADR and PDR. A recent meta-analysis found that patients who had received enhanced instructions (social media apps, SMS, and telephone calls) had higher cecal intubation rates (odds ratio 2.77, 95% CI 1.73-4.42; *P*<.001) than patients receiving regular verbal and written instructions [4]. In this study, none of the cases in which the cecum was not reached were because of inadequate bowel preparation, although it has been reported as a major factor in the literature [44].

Bowel preparation procedures may cause discomfort. The main discomfort patients report relates to uncertainties with respect to dietary recommendations and adverse gastrointestinal symptoms owing to use of laxatives [33]. Patient education using a smartphone app may help resolve these uncertainties [45]. Indeed, the willingness to repeat the preparation procedure was higher for patients receiving enhanced bowel preparation instructions than for those receiving regular instructions (odds ratio 1.91, 95% CI 1.20-3.04; *P*=.01) [4]. High patient satisfaction can therefore help to increase patient participation in surveillance colonoscopies. In our control group, patient satisfaction was already high and increased further when using the smartphone app.

### Strengths and Limitations

This study had several strengths. Selection bias was avoided in three ways. First, inclusion concerned screening, surveillance, and symptomatic patients of both morning and afternoon colonoscopies. Second, patients were not excluded if they had a history of abdominal surgery, diverticulosis, stenosis, or constipation, compared with most other studies [2,22,31,35,46]. Third, the app was available for smartphones with both Android and iOS operating systems, in contrast to the study by Lorenzo-Zuniga et al [36]. Furthermore, no maximum age for participation was stated. All the abovementioned decisions in the methodology add to the generalizability of our findings.

This study also had certain limitations. First, compliance with the bowel preparation schedule was not controlled in either group, although it is known that approximately 30% of patients with poor bowel preparation fail to follow instructions before the colonoscopy [23]. In addition, we did not monitor other variables related to BBPS, such as searching for additional information on the internet or other social media or help provided by other sources or people. Second, the patients were not blinded to the intervention. Third, a large number of endoscopists assessed the BBPS, potentially leading to a larger variability in scoring and possibly causing bias. All endoscopists were trained and experienced in using the BBPS to achieve uniform scoring, thereby reflecting daily endoscopic practice in a teaching hospital. Fourth, selection bias may have occurred, as only 30.8% (180/584) of the screened patients visiting our prescreen facility were eligible for inclusion. Most likely, only patients with an affinity for smartphone use were willing to participate, lowering the generalizability of this study. With the expectation of an increase in smartphone use in the future, generalizability will subsequently increase, and smartphone apps for bowel preparation can be a valuable tool in improving bowel preparation quality. Fifth, the study was performed at a single center, limiting its generalizability.

### Conclusions

In conclusion, this study showed that using our personalized smartphone app significantly improved bowel preparation quality, particularly in the right colon, and could improve polyp detection in the right colon. Patient satisfaction was equal in the personalized smartphone app group and the control group. Smartphone apps are an easy-to-use tool to improve patients’ bowel preparation education and quality, making implementation in clinical practice feasible.

## Appendix

Multimedia Appendix 1 CONSORT-eHEALTH checklist (V 1.6.1).

Multimedia Appendix 2 eHealth checklist patient satisfaction questionnaire-18.

## Abbreviations

ADR: adenoma detection rate
BBPS: Boston Bowel Preparation Scale
PDR: polyp detection rate
PSQ-18: Patient Satisfaction Questionnaire-18
SPMC: sodium picosulfate with magnesium citrate
