# Supplementary material for: Effects of a Personalized Smartphone App on Bowel Preparation Quality: Randomized Controlled Trial
Source: JMIR Mhealth Uhealth. 2021 Aug 19;9(8):e26703. doi: 10.2196/26703 (PMC8414298; doi:10.2196/26703)
Supplement: Multimedia Appendix 2 [file mhealth_v9i8e26703_app2.docx]

## MULTIMEDIA APPENDIX OF SUPPLEMENTARY FILES

**Patient satisfaction questionnaire (PSQ-18).**

Questions concerning the care organized surrounding the colonoscopy, for patients in both the smartphone and control group (on a 5-point Likert scale):

1. It was properly explained to me why a colonoscopy was necessary;
2. I was able to ask all the questions I wanted to ask;
3. I am dissatisfied about some aspects of the information I received about the procedure;
4. I have been able to use the bowel preparation the way I was explained to do;
5. I think my bowel is sufficiently clean on the day of the procedure;
6. The information I received cannot be improved;
7. Enough time has been spent to explain the procedure;
8. All my personal details were accurately checked during the screening visit;
9. In my opinion, this hospital has everything that is necessary for my colonoscopy.

Questions concerning the smartphone application, solely for patients in the smartphone group:

(on a 5-point Likert scale)

1. The application was easy to download and use;
2. It was clear at what times I had to take the bowel preparation laxative;
3. I think the application adds value to the regular instructions;
4. For a possible new colonoscopy in the future, I would use the application again.

(on a 10-point Likert scale):

1. How do you rate the user-friendliness of the application in general;
2. How do you rate the (visual) design of the application?
